# Editorial: Modulation of T-cell function and survival by the tumor microenvironment

**DOI:** 10.3389/fcell.2023.1271772

**Published:** 2023-08-24

**Authors:** Kristian M. Hargadon, Courtney M. Lappas, Shahrzad Jalali

**Affiliations:** ^1^ Department of Biology, Hampden-Sydney College, Hampden-Sydney, VA, United States; ^2^ Department of Biology, Lebanon Valley College, Annville, PA, United States; ^3^ Department of Oncology, Mayo Clinic, Rochester, MN, United States

**Keywords:** cancer, tumor microenvironment, T cell, immune regulation, tumor immunity

T lymphocytes are widely recognized as critical regulators of anti-tumor immune surveillance, and advances in our understanding of these cells in the context of cancer over the last two decades have sparked a rapid growth in T cell-based immunotherapies to treat the disease. From therapeutic vaccines that aim to elicit responses by otherwise dormant tumor-specific T lymphocytes, to adoptive cell transfer therapies that harness the anti-tumor potential of both natural and antigen-redirected T cells, to checkpoint blockade regimens that unleash the full power of these cells in their fight against cancer, the era of immuno-oncology has brought with it a dramatic change in the landscape of therapeutic options available for patients. At the same time, although these approaches have yielded response rates and clinical outcomes that are unprecedented in the history of cancer therapy, there remain significant challenges that often limit the reach and durability of T lymphocyte-based immunotherapies.

One challenge that has emerged as a particularly significant barrier to immunotherapy is the immunologically hostile nature of the tumor microenvironment (TME), a complex ecosystem comprised of diverse cell types (tumor cells, stromal cells, immune cells, fibroblasts, adipocytes, vascular endothelial cells, and microbes) and non-cellular components (metabolites, cytokines, extracellular matrix, exosomes, and other cell-derived factors) that evolves to support the dynamic needs of cancer cells over the course of tumor progression. The adaptation of cancer cells to not only survive in, but also thrive in, an ecological niche that at the same time promotes anti-tumor T cell dysfunction is indeed a driving force behind tumor immune escape.

In this Research Topic, we present a collection of original research and review articles from leading experts that highlight many of the complex interactions between T lymphocytes and the TME. These articles document the diverse ways in which the TME is now known to modulate T cell function and survival, and they highlight how interventions that aim to disrupt the immunosuppressive networks within this internal ecosystem can support more favorable outcomes of anti-tumor immune responses. Importantly, this collection also brings focus to important issues that remain to be addressed in this field as we seek to further improve the efficacy of immune-based interventions for cancer.

Our Research Topic opens with a comprehensive review by Mani et al., who describe various immune-modulating factors within the TME that compromise T cell functionality. Particular attention is given to the role of regulatory T cells (Tregs) and myeloid-derived suppressor cells (MDSC) in dampening T cell proliferation and effector function, oncometabolites and other metabolic constraints that inhibit anti-tumor T cell reactivity, and immune checkpoint ligands that promote T cell exhaustion. Emphasis is also placed on inflammatory mediators and chemokines within the TME that act as key regulators of T lymphocyte-vascular endothelial cell interactions necessary for effector T cell entry and infiltration into the TME. A deeper discussion of T lymphocyte suppression by MDSC is then provided by Bhardwaj and Ansell, who focus on the influence of these immunoregulatory cells specifically within the context of hematologic malignancies, and Qi et al. bring focused attention to the immunosuppressive functions of Tregs, with particular emphasis on the role of the CBM complex/NF-κB, MAPK/CDK, and STAT3/P27 signaling pathways in these cells as contributing factors to TME formation and intratumoral Treg function.

We next present two review articles that highlight how non-immune cell populations and their products can influence the function of T lymphocytes within the TME. DiPalma and Blattman discuss the regulation of intratumoral T cell function by the tumor microbiome as well as distant microbiomes (such as the gut microbiome), highlighting the roles of specific microbial species, microbe-derived metabolites, and microbial dysbiosis in shaping both the TME and anti-tumor T cell reactivity at this site. Their article concludes with a discussion of recent insights from murine and clinical studies that have shed light on microbial dysbiosis and dietary influences on microbiome composition as determinants of therapeutic efficacy for adoptive cell transfer and checkpoint blockade regimens. Whiteside then reviews the mechanisms by which melanoma-derived exosomes released from the TME can suppress effector function and induce apoptosis of T cells even prior to tumor infiltration.

Among tumors that have been successfully infiltrated by T lymphocytes, a common phenomenon associated with loss of immunologic control and tumor progression is T cell exhaustion. Arising from chronic antigenic exposure and engagement of various immune checkpoints within the TME, T cell exhaustion is associated with reduced proliferative capacity, anti-tumor reactivity, and persistence of T cells within tumor tissue. Our Research Topic offers two excellent reviews on this Research Topic. First, Blake et al. describe epigenetic regulation of various T cell exhaustion phenotypes that have been reported in the TME, highlighting the role of both transcription factors and chromatin modifiers known to program the exhausted state. They also discuss epigenetic modulators that are currently being explored as a means of rewiring T cells for more robust anti-tumor reactivity. This review is followed by a more specific assessment of exhaustion in the context of CAR-T cell therapy. Zhu et al. summarize the various factors known to influence CAR-T cell exhaustion, from the design of CAR-T receptors, to *in vitro* expansion conditions during CAR-T cell preparation, to regulatory effects of the TME on CAR-T cell functionality. They conclude by discussing combinatorial strategies for targeting immunosuppressive factors within the TME as a means of preventing the exhaustion of CAR-T cells at this site.

Lastly, we close our Research Topic with two original research articles that have important implications for T lymphocyte-based cancer immunotherapy. Gamache et al. demonstrate in an orthotopic mouse model of pancreatic adenocarcinoma that CD40 stimulation remodels the TME to support more robust anti-tumor immunity. Notably, CD40 agonism in combination with checkpoint blockade led to a reduction in both conventional CD4^+^ Tregs as well as CD8^+^ Tregs within the TME, and this change was associated with enhanced T cell effector function, improved responses to checkpoint blockade therapy, and establishment of CD4-dependent immunologic memory. CD4^+^ T cells within the TME are also the focus of a prognostic gene expression index for gastric cancer developed by Chen et al. Among CD4^+^ T cell-related hub genes analyzed, *PROC* and *SERPINE1* were found to carry prognostic significance for gastric cancer and were used to construct a risk-score that correlated with patient outcome and tumor infiltration by CD8^+^ T cells. As such, this risk-score model may serve as a useful predictor for gastric cancer responsiveness to immunotherapy.

As the era of immuno-oncology continues to unfold at a rapid pace, there is indeed a renewed optimism for the future of cancer care. Though the TME remains a daunting barrier to the success of many immunotherapies, advances in our understanding of this dynamic structure have reshaped the way we think about tumors and tumor immunity. Rather than a simple T cell versus tumor cell battleground, the TME is now appreciated to be a unique and complicated ecological niche, with a multitude of cellular and molecular interactions ultimately influencing the outcome of the anti-tumor immune response. Insights into these relationships within the TME are now opening up exciting opportunities to reshape this ecosystem in ways that support more robust tumor immune reactivity. With this goal in mind, it is our hope that the articles presented herein not only shed light on the complexities of the TME but also spark future research efforts to further our understanding of T cell regulation in the TME and improve treatment outcomes for cancer immunotherapy in the years ahead.

